# uShuffle: A useful tool for shuffling biological sequences while preserving the k-let counts

**DOI:** 10.1186/1471-2105-9-192

**Published:** 2008-04-11

**Authors:** Minghui Jiang, James Anderson, Joel Gillespie, Martin Mayne

**Affiliations:** 1 Department of Computer Science, Utah State University, Logan, UT 84322-4205, USA

## Abstract

**Background:**

Randomly shuffled sequences are routinely used in sequence analysis to evaluate the statistical significance of a biological sequence. In many cases, biologists need sophisticated shuffling tools that preserve not only the counts of distinct letters but also higher-order statistics such as doublet counts, triplet counts, and, in general, *k*-let counts.

**Results:**

We present a sequence analysis tool (named uShuffle) for generating uniform random permutations of biological sequences (such as DNAs, RNAs, and proteins) that preserve the exact *k*-let counts. The uShuffle tool implements the latest variant of the Euler algorithm and uses Wilson's algorithm in the crucial step of arborescence generation. It is carefully engineered and extremely efficient. The uShuffle tool achieves maximum flexibility by allowing arbitrary alphabet size and let size. It can be used as a command-line program, a web application, or a utility library. Source code in C, Java, and C#, and integration instructions for Perl and Python are provided.

**Conclusion:**

The uShuffle tool surpasses existing implementation of the Euler algorithm in both performance and flexibility. It is a useful tool for the bioinformatics community.

## Background

Randomly shuffled sequences are routinely used in sequence analysis to evaluate the statistical significance of a biological sequence. For example, a common method for assessing the thermodynamic stability of an RNA sequence is to compare its folding free energy with those of a large sample of random sequences. It is known that the stability of an RNA secondary structure depends crucially on the stackings of adjacent base pairs; therefore the frequencies of distinct doublets in the random sequences are important considerations in such analysis [4,25]. Besides, natural biological sequences often manifest certain nearest-neighbor patterns: both eukaryotic and prokaryotic nucleic acid sequences show a consistent hierarchy in the doublet frequencies; in coding regions, the codon usage can also be markedly nonuniform. In many cases, biologists need sophisticated shuffling tools that preserve not only the counts of distinct letters but also higher-order statistics such as doublet counts, triplet counts, and, in general, *k*-let counts.

### Methods for random sequence generation

Several methods are commonly used to generate random sequences. The *basic permutation method *works as follows: for a sequence *S *[1, *n*], pick a random number *i *between 1 and *n*, swap the two elements *S *[*i*] and *S *[*n*], then recurse on the subsequence *S *[1, *n *- 1]. The random sequence generated by the basic permutation method preserves the exact count of each distinct letter in the alphabet, but does not preserve the higher-order statistics of *k*-let counts. The *Markov method *[12], which is based on the Markov chains, generates random sequences that preserve the *k*-let counts only on average: the counts of the individual sequences may deviate from the input distribution. The *swapping method *[15], a popular method which is now folklore, generates random sequences by repeatedly swapping disjoint subsequences flanked by the same (*k *- 1)-lets; it does preserve the *k*-let counts exactly, but produces random sequences that are only uniform asymptotically and may need a large number of swapping steps.

### The Euler algorithm preserves exact *k*-let counts

The Euler algorithm is a less-known but very efficient algorithm for generating truly uniform random *k*-let-preserving sequences [2,12,15]. We briefly review its history. Fitch [12] first noticed that a doublet-preserving permutation is related to an Eulerian walk of a directed multigraph; however, the algorithm he proposed does not generate all permutations with equal probability. Altschul and Erickson [2] presented the first algorithm (also based on Eulerian walks in directed multigraphs) for generating truly uniform random sequences that preserve either the doublet counts or the triplet counts or both; however, a crucial step of their algorithm for generating random arborescences depends on a trial-and-error procedure, which is a potential bottleneck in performance. This bottleneck was eliminated by Kandel et al. [15], who replaced the trial-and-error procedure with a simple and efficient procedure based on random walks in directed multigraphs. They also generalized the Euler algorithm to preserve the *k*-let counts for arbitrary *k*, and suggested a simple data structure for implementation. This data structure is based on look-up tables and requires *O*(*σ*^2*k*-2^) space and time; it quickly become inefficient as the alphabet size *σ *and the let size *k *increase. Since the work by Kandel et al. [15], a better algorithm has been proposed by Wilson [19,23] for generating random arborescences, which is the crucial step of the Euler algorithm that Kandel et al. [15] improves upon Altschul and Erickson [2]. The superiority of Wilson's arborescence generation algorithm to the two previous algorithms by Altschul and Erickson [2] and by Kandel et al. [15] is both proved in the theoretical sense by Wilson [19,23], and demonstrated in the practical sense by a comparison of our implementation with a previous implementation (to be discussed later).

### Implementations of the Euler algorithm

We are aware of two previous implementations of earlier variants of the Euler algorithm. The *dishuffle *program by Clote et al. [6] implements the original version of the Euler algorithm by Altschul and Erickson [2]. The *shufflet *program by Coward [11] implements the improved version of the Euler algorithm by Kandel et al. [15]. In this paper, we present a sequence analysis tool (named uShuffle) for shuffling biological sequences while preserving the *k*-let counts. The uShuffle program is based on the latest variant of the Euler algorithm [2,15] and uses Wilson's algorithm [19,23] in the crucial step of arborescence generation. Our goal is to provide a versatile tool that is as efficient and as flexible as possible:

#### Arbitrary alphabet size and let size

In specific applications, the alphabet size *σ *and the let size *k *are often fixed: for biological sequences, typical alphabet sizes are 4 (for DNAs or RNAs) and 20 (for proteins), and typical let sizes are 2 (for dinucleotides) and 3 (for codons). While it is tempting to implement the Euler algorithm just for the fixed alphabet and let sizes at hand, we believe the flexibility of arbitrary alphabet and let sizes is useful. The dishuffle program by Clote et al. [6], for example, is hard-coded for shuffling RNA sequences preserving dinucleotide counts (with alphabet size *σ *= 4 and let size *k *= 2). It is apparent that such an implementation cannot be used easily in other applications with different alphabet and let sizes.

#### Efficiency

When the alphabet size and the let size are both small constants, the running time of the Euler algorithm (with any of the three variants of arborescence generation [2,15,23]) is linear in the sequence length. So it may appear that the efficiency of the shuffling program would not an issue since any conceivable downstream analysis of the randomized data would much slower than the shuffling. However, we note that the linear running time has been proved only for the case that the alphabet and let sizes are constant [15]. It is not at all clear whether the linear performance of the Euler algorithm is scalable for arbitrary alphabet and let sizes. As mentioned earlier, the "standard" data structure suggested by Kandel et al. [15] has time and space complexities *O*(*σ*^2*k*-2^), which can become exponential when the alphabet size *σ *and the let size *k *become large, approaching the order of the sequence length. Indeed, as we will discuss later, we have reason to believe that this very data structure has been used in the shufflet program by Coward [11].

Furthermore, the implementation of the Euler algorithm (in particular, the crucial step of arborescence generation) is non-trivial because of its heavy use of graph-theoretical concepts such as directed multigraphs and Eulerian walks. Although Wilson's celebrated algorithm [19,23] dates back to 1996, and is well-known in the theoretical computer science community, Coward's implementation of shufflet in 1999 [11] still uses the old arborescence algorithm by Kandel et al. [15]. We are not aware of any implementation of Wilson's algorithm in bioinformatics applications. By careful choices of algorithms and data structures, and by scrupulous algorithmic engineering, we strive for the most efficient implementation.

#### Multiple forms and programming languages

The dishuffle program by Clote et al. [6] is written in Python; the shufflet program by Coward [11] is a web application in C. To reach the widest audience, we have made our uShuffle program available in several forms. It can be used as a command-line program, a web application, or a utility library. Source code in C, Java, and C#, and integration instructions for Perl and Python are provided.

## Implementation

This section consists of four subsections. In the first two subsections, we discuss, at a conceptual level, the Euler algorithm and its crucial step of arborescence generation, in preparation of the discussion of implementation details. In the third subsection, we present the algorithmic engineering details of our implementation. In the fourth subsection, we describe the software organization and user interfaces of the uShuffle tool. To justify our algorithm choices and to explain our optimization techniques, the discussions in the first three subsections are necessarily technical. The readers who are not particularly interested in the theoretical discussion of graph algorithms or the technical details of algorithmic engineering can safely skip to the fourth subsection for the software organization and user interfaces.

### The Euler Algorithm

In this subsection, we review some basic concepts of the Euler algorithm.

#### Directed multigraph

**Figure 1:**
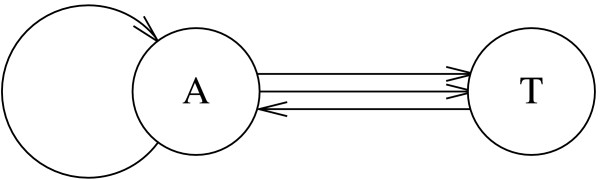
Directed Multigraph for the Sequence AATAT.

A *k*-let is a subsequence of *k *consecutive elements in a sequence. Let *S *be a sequence to be permuted. Let *T*_*k *_be a uniform random sequence that preserves the *k*-let counts of *S*. (For example, *T*_1 _is a simple permutation of *S*, and *T*_2 _is a permutation of *S *with the same dinucleotide counts.) To generate *T*_*k *_for *k *≥ 2, the Euler algorithm [2,15] first constructs a directed multigraph *G*. We refer to Figure 1 for an example. For each distinct (*k *- 1)-let in *S*, *G *has a vertex. For each *k*-let *L *in *S*, which contains two (*k *- 1)-lets *L*_1 _and *L*_2 _such that *L*_1 _precedes *L*_2_, *G *has a directed edge from the vertex for *L*_1 _to the vertex for *L*_2_. Duplicates of *k*-lets may exist in *S*, so there may be multiple edges between the vertices.

#### Correspondence between permutations and Eulerian walks

As we scan the *k*-lets in *S *one by one, we also walk in the directed multigraph *G *from vertex to vertex. When all the *k*-lets are scanned, each edge in *G *is visited exactly once: the walk is *Eulerian*. On the other hand, given an Eulerian walk in *G*, we can recover a sequence by spelling out the (*k *- 1)-lets of the vertices along the walk (and discarding the overlaps). Since each *k*-let in *S *corresponds to an edge in *G*, every Eulerian walk in *G *corresponds to a sequence with the same *k*-let counts as *S*. Kandel et al. [15] showed that, as long as an Eulerian walk starts and ends at the same two vertices *s *and *t *that correspond to the starting and the ending (*k *- 1)-lets of *S*, the *i*-let counts for all 1 ≤ *i *≤ *k *are preserved. Therefore, generating a uniform random sequence *T*_*k *_reduces to generating a uniform random Eulerian walk in *G *from *s *to *t*.

#### Correspondence between Eulerian walks and arborescences

For an Eulerian walk in *G*, each vertex *v *of *G *except the ending vertex *t *has a *last edge e*_*v *_that exits from *v *for the last time. The set of last edges for all vertices except *t *forms an *arborescence *rooted at *t*: a directed spanning tree in which all vertices can reach *t*. Given an arborescence *A *rooted at *t*, a random Eulerian walk from *s *to *t *with the last edges conforming to *A *can be easily generated [2,15]:

1. For each vertex *v*, collect the list of edges *E*_*v *_exiting from *v*. Permute each edge list *E*_*v *_separately while keeping *e*_*v *_the last edge on the list.

2. Walk the graph *G *in accordance with the edge lists {*E*_*v*_}: start from *s *(set *u *← *s*), take the first unmarked edge (*u, v*) from the list *E*_*u*_, mark the edge, then move to the next vertex *v *(set *u *← *v*); continue until all edges are marked and the walk ends at *t*.

In directed multigraphs, there is a nice correspondence between Eulerian walks and arborescences: every arborescence rooted at *t *corresponds to exactly the same number of Eulerian walks [3,15]. Therefore, generating a uniform random Eulerian walk in *G *from *s *to *t *reduces to generating a uniform random arborescence in *G *rooted at *t*. In the next subsection, we discuss algorithms for generating random arborescences, some of which are based on, quite amusingly, random walks again.

### Generating Random Arborescences

In this subsection, we review the existing algorithms for arborescence generation, and explain our choice of Wilson's algorithm [19,23]. There are two major approaches to generating random arborescences and spanning trees: determinant algorithms and random-walk algorithms.

#### Determinant algorithms

Determinant algorithms are based on the matrix tree theorem [3, Chapter II, Theorem 14]. For a graph *G*, the probability that a particular edge *e *appears in a uniform random spanning tree is the ratio of two numbers: the number of spanning trees that contain the edge *e*, and the total number of spanning trees. The matrix tree theorem allows one to compute the exact number of spanning trees of a graph by evaluating the determinant of the combinatorial Laplacian (or Kirchhoff matrix) of the graph. A random spanning tree can be generated by repeatedly contracting or deleting edges according to their probabilities.

The first determinant algorithms were given by Guénoche [14] and Kulkarni [16]: for a graph of *n *vertices and *m *edges, a random spanning tree can be generated in *O*(*n*^3^*m*) time. This running time was later improved to *O*(*n*^3^) [7]. Colbourn, Myrvold, and Neufeld [8] simplified the *O*(*n*^3^) time algorithm and showed that the running time can be further reduced to *O*(*n*^2.376^), the best upper bound for multiplying two *n *× *n *matrices [9].

#### Random-walk algorithms

Random-walk algorithms use an entirely different approach to generating random spanning trees. Aldous [1] and Broder [5] (after discussing the matrix tree theorem with Diaconis) independently discovered an interesting connection between random spanning trees and random walks:

Simulate a uniform random walk in a graph *G *starting at an arbitrary vertex *s *until all vertices are visited. For each vertex *v *≠ *s*, collect the edge {*u, v*} that corresponds to the first entrance to *v*. The collection *T *of edges is a uniform random spanning tree of *G*.

For a graph *G *and a vertex *v *in it, define the *cover time C*_*v*_(*G*) as the expected number of steps a random walk starting from *v *takes to visit all vertices of *G*. The running time of the Aldous-Broder algorithm [1,5] is clearly linear in the cover time. In the context of shuffling biological sequences, Kandel et al. [15] extended Aldous-Broder algorithm [1,5] to generate uniform random arborescences of Eulerian directed graphs in the cover time. Wilson and Propp [24] then presented an algorithm for generating uniform random arborescences of general directed graphs in 18 cover times.

#### Wilson's algorithm

Wilson [19,23] showed that random arborescences and spanning trees can be generated more quickly than the cover time by a cycle-popping algorithm which simulates loop-erased random walks. For a graph *G *and two vertices *u *and *v *in it, define the *hitting time h*_*u*,*v*_(*G*) as the expected number of steps a random walk takes from *u *to *v*. The running time of Wilson's algorithm [19,23] is linear in the maximum or mean hitting times of the corresponding stochastic graphs. As Wilson [19,23] noted, the mean and maximum hitting times are always less than the cover time, and the differences can be quite significant in certain graphs. Therefore, for generating uniform random arborescences, Wilson's algorithm [19,23] is superior to Kandel et al.'s algorithm [15].

For completeness of presentation, we include in the following the pseudocode of Wilson's algorithm [19,23]:

RandomTreeWithRoot(*r*)

1   for *i *← 1 to *n*

2      *InTree *[*i*] ← false

3   *Next *[*r*] ← nil

4   *InTree *[*r*] ← true

5   for *i *← 1 to *n*

6      *u *← *i*

7      while not *InTree *[*u*]

8         *Next *[*u*] ← RandomSuccessor(*u*)

9          *u *← *Next *[*u*]

10      *u *← *i*

11      while not *InTree *[*u*]

12         *InTree *[*u*] ← true

13         *u *← *Next *[*u*]

14   return *Next*

Let *E*_*u *_be the set of directed edges exiting from the vertex *u*. The function RandomSuccessor(*u*) chooses a uniformly random edge (*u, v*) from *E*_*u*_, then returns the vertex *v*.

Unlike the Aldous-Broder algorithm [1,5], which simulates a single random walk from the root to visit all vertices, Wilson's algorithm [19,23] simulates multiple random walks: starting from each unvisited vertex, a random walk continues until it joins a growing arborescence which initially contains only the root. A random walk follows the *Next *[·] pointers; whenever a previously visited vertex is encountered again, a loop is formed and immediately erased because the *Next* [·] pointer is overwritten (in the first while loop). As soon as a walk reaches the growing arborescence, all vertices in the walk join the arborescence as one more branch.

#### A comparison of the two approaches

We now give a comparison of the two approaches to generating random arborescences. Kandel et al. [15] proved that the cover time of an Eulerian directed multigraph of *n *vertices and *m *edges is *O*(*n*^2^*m*). From our preceding discussion on the cover time and the hitting time, it follows that the expected running time of Wilson's algorithm [19,23] on the same multigraph is at most *O*(*n*^2^*m*) too, neglecting the *log n* factors.

For a multigraph, the number *m *of edges can be arbitrarily larger than the number *n *of vertices. So it might appear that the determinant algorithm by Colbourn et al. [8], which runs in deterministic *O*(*n*^3^) time or even *O*(*n*^2.376^) time, would be a better alternative than the random walk algorithms [15,19,23]. However, we note that when *m *is large the intermediate values of the determinant computation can be large too. On the typical computer systems today, the arithmetic operations on floating-point numbers do not have enough precision to guarantee the accuracy and stability in the numerical computation of the determinant algorithms. The random walk algorithms [15,19,23], on the other hand, require only basic operations on small integers, and do not have these numerical problems. Therefore, we have decided to implement Wilson's random-walk algorithm [19,23] for arborescence generation.

### Implementation Details

In this subsection, we describe the details of our implementation of the Euler algorithm [2,15,19,23] for generating *k*-let-preserving random sequences.

#### Kandel et al.'s data structure

As suggested by Kandel et al. [15], a simple implementation of the Euler algorithm [2,15] may use a look-up table of size *σ*^*k*-1 ^for all possible (*k *- 1)-lets as vertices in the directed multigraph *G*, then build an adjacency matrix of size *σ*^*k*-1 ^× *σ*^*k*-1 ^for the edges in *G*. When both *σ *and *k *are small constants, the space requirement of this simple approach, *σ*^2*k*-2^, may not look severe. However, a calculation shows that, even for *σ *= 20 (the alphabet size of proteins) and *k *= 3 (a typical choice of *k*), the space requirement amounts to

*σ*^2*k*-2 ^= 20^4 ^= 160, 000.

On the other hand, the typical length of a protein sequence is below 1000. Even though a sequence itself may be stored in only 1 kilo-bytes, the permutation algorithm still requires hundreds of times more space regardless. The situation becomes even worse when *k *is further increased: even for the rather innocent-looking parameters *σ *= 20 and *k *= 5, the space requirement

*σ*^2*k*-2 ^= 20^8 ^> 16^8 ^= 2^32^

exceeds all 4 giga-bytes of memory that can be accommodated by a 32-bit computer! We note that the two sets of parameters that Coward [11] used for experiments on his shufflet program were only

*σ *= 4, *k *= 6, *σ*^2*k*-2 ^= 1, 048, 576

and

*σ *= 20, *k *= 3, *σ*^2*k*-2 ^= 160, 000.

We will discuss more about this in our comparison of uShuffle and shufflet in the Results and Discussion section.

#### Representing directed multigraph in linear space

To make the uShuffle program scalable, it is clear that careful algorithmic engineering are necessary in the implementation. As we discussed in the previous subsection on the Euler algorithm, the directed multigraph *G *contains a vertex for each distinct (*k *- 1)-let in *S*. Since the number of (*k *- 1)-lets in *S *is exactly *l *- *k *+ 2, *G *has at most *l *- *k *+ 2 vertices, and hence exactly *l *- *k *+ 1 directed edges between consecutive (*k *- 1)-lets. This implies that the size of *G *is in fact linear in the length *l *of the sequence *S *to be permuted. With suitable data structures, uShuffle needs only linear space.

In the following, we first explain the construction and representation of the directed multigraph *G*, then explain the random sequence generation after the graph construction. The graph construction consists of two steps: determine the set of vertices, then add the directed edges.

#### Determining vertices

We use a hashtable to determine the set of vertices. The hashtable consists of a bucket array of size *b *= *l *- *k *+ 2, the number of (*k *- 1) lets in *S*, and a linked list at each bucket to avoid collision by chaining [10]. Each (*k *- 1)-let *x *= *x*_1_*x*_2_⋯*x*_*k*-1 _has a polynomial hash code

$$\begin{array}{lll} h(x) & = & x_{1}a^{k-1}+x_{2}a^{k-2}+\cdots +x_{k-2}a^{2}+x_{k-1}a\\ & = & ((\cdots (x_{1}a+x_{2})a+\cdots +x_{k-2})a+x_{k-1})a, \end{array}$$

where *a *= $\left(\sqrt{5}-1\right)$/2 is the reciprocal of the golden ratio; the index of *x *to the bucket array is

*i*(*x*) = ⌊*h*(*x*)·*b*⌋ mod *b*.

Initialize the hashtable to be empty, then try to insert the (*k *- 1)-lets into the hashtable one by one. If a (*k *- 1)-let is the first of its kind, it is assigned a new vertex number then inserted into the hashtable; its starting index to the sequence *S *is also recorded. If a (*k *- 1)-let has been inserted before, it is not inserted to the hashtable: its vertex number and index to the sequence *S *are copied from those of the first (*k *- 1)-let of its kind. After insertions, we can deduce the total number of vertices in the directed multigraph from the largest vertex number assigned. The memory for vertices are then allocated.

#### Adding directed edges

To add the directed edges, we use an adjacency-list representation to avoid the excessive memory requirement of an adjacency-matrix. In an adjacency-list representation, two edge lists need to be maintained at each vertex: a list of incoming edges and a list of outgoing edges. The outgoing edge lists are necessary for generating Eulerian walks [2]. The incoming edges lists are necessary for generating arborescences when Kandel et al.'s algorithm [15] is used (as in the implementation by Coward [11]). We use Wilson's algorithm [19,23] for generating arborescences. As we discussed in the previous section, Wilson's algorithm [19,23] is faster than Kandel et al.'s algorithm [15]. Furthermore, we note here that Wilson's algorithm [19,23] has another advantage over Kandel et al.'s algorithm [15] in terms of the ease of implementation. Instead of one *backward *random walk from the ending vertex *t *to reach all other vertices as in Kandel et al.'s algorithm [15], Wilson's algorithm [19,23] uses multiple *forward *random walks from each unvisited vertex to join the arborescence rooted at *t*: the outgoing edge lists alone are sufficient for generating both the Eulerian walks and the arborescences.

#### Representing edge lists and managing memory

For maximum efficiency, we implement each edge list as an array of vertices. The numbers of outgoing edges differ from vertex to vertex; if we allocate a fixed-size array for each vertex, then we would have to make each array large enough to hold all edges in the worst case, and the resulting space requirement would become quadratic in the length *l *of the sequence *S*. We could of course first count the number of outgoing edges for each vertex, then allocate a separate array just large enough for each vertex. However, this would require us to call the relatively expensive memory allocation function once for each vertex.

In our implementation, we allocate one large array for all edges (the total number of edges is *l *- *k *+ 1), then parcel out pieces to individual vertices. To achieve this, we first scan the sequence *S *to count the number of outgoing edges for each vertex, then point the array (outgoing edge list) of each vertex to successive offsets of the large array. With this optimization, the number of memory allocations is reduced to only 4: one for the hashtable bucket array, one for the array of (*k *- 1)-lets as hashtable entries, one of the array of vertices, and one for the array of edges. The memory for the bucket array and the hashtable entries can be freed as soon as the directed multigraph is constructed.

#### Sequence generation after graph construction

After the construction of the directed multigraph, we can generate a random sequence in three steps. As discussed in the previous section, we need to first simulate the loop-erased random walks [19,23] to generate an arborescence, next permute the individual edge lists while maintaining the last edges, then simulate an Eulerian walk guided by the edge lists and output the sequence along the walk. Since each edge list is implemented as an array, the permutation can be executed very efficiently. To output the random sequence along the walk is also easy, since each vertex keeps the starting index of its first occurrence in the input sequence.

### Software Organization and User Interfaces of the uShuffle Tool

In this subsection, we describe the software organization and user interfaces of the uShuffle tool.

#### C Library and command-line tool

Our initial implementation of uShuffle is in the C programming language. The C version of uShuffle consists of two components: a uShuffle library (ushuffle.c and ushuffle.h) and a command-line tool (main.c).

In a typical scenario, multiple *k*-let-preserving random sequences are generated for each input sequence. The graph construction stage of the uShuffle program needs to be done only once for the multiple output sequences. To give the users an option for optimization, we export three interface functions in the uShuffle library:

void shuffle(const char *s, char *t, int l, int k);

void shuffle1(const char *s, int l, int k);

void shuffle2(char *t);

The function shuffle accepts four parameters: s is the sequence to be permuted, t is the output random sequence, l is the length of s, and k is the let size *k*. The function shuffle simply calls shuffle1 first and shuffle2 next: shuffle1 implements the construction of the directed multigraph; shuffle2 implements the loop-erased random walks in the directed multigraph and the generation of the random sequence. The statistical behavior of a random permutation depends heavily on the random number generator.

Coward [11] noted that the default implementations of random number generators on various platforms are often unsatisfying, so he implemented his own generator using an arguably better algorithm. We note that there are numerous algorithms for random number generation, and new algorithms are continuously being proposed: whether one algorithm is superior to the other can be quite subjective. Instead of limiting the users to a particular implementation, we set the default generator to the random function from the standard C library, then export an interface function to allow sophisticated users to customize the generator:

typedef long (*randfunc_t)();

void set_randfunc(randfunc_t randfunc);

The command-line uShuffle tool is a minimal front-end of the uShuffle library that illustrates a typical use of the library. It has the following four options:

-s <string> specifies the input sequence,

-n <number> specifies the number of random sequences to generate,

-k <number> specifies the let size,

-seed <number> specifies the seed for random number generator.

#### Java applet

**Figure 2:**
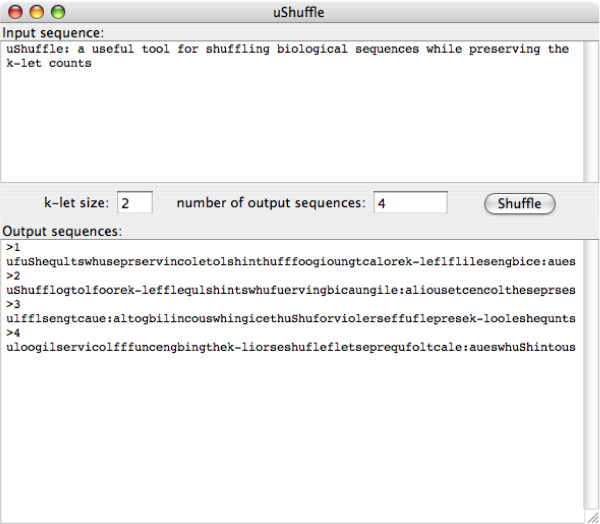
Screenshot of uShuffle Java Applet.

The uShuffle program is ported to the Java programming language. Beside having a library and a command-line tool, the Java version of the uShuffle program can also run as an applet in a web browser. We refer to Figure 2 for a screenshot of the uShuffle Java applet^1^: The interface of the applet is minimal and consists of three parts: an input text area at the top, an output text area at the bottom, and a control panel in the middle. The control panel contains two text fields and a button. The maximum let size *k *and the number *n *of output sequences can be set in the two text fields. When the "Shuffle" button is clicked, the applet takes the input sequence from the input text field, strips away the white spaces, generates *n *random sequences that preserve the *k*-let counts, then outputs the sequences in the output text area. The output is in the Fasta format when *n *> 1: each output sequence is preceded by a comment line containing a sequence number ranging from 1 to *n*.

The uShuffle Java applet keeps all the output sequences in memory for display in the output text area. When the number *n *of output sequences and the input sequence length *l *are exorbitantly large, for example, *n *= 10, 000, 000 and *l *= 100, the total memory required to hold the output sequences may exceed the maximum heap size of the Java virtual machine (JVM) and the applet may hang. This is not a bug in our program but is due to the limit of JVM; nevertheless, we prepared a web page to instruct the users how to increase the maximum heap size of JVM.

#### C#/Perl/Python versions

The uShuffle program is also ported to the C# programming language. Perl and Python are popular programming languages for bioinformatics; they allow easy integration with programs written in C. Instead of porting the uShuffle program to Perl and Python at the source code level, we prepared two web pages to instruct the users how to extend the Perl and Python environments with the uShuffle library.

## Results and Discussion

We have performed two sets of experiments to test the performance of two major forms of the uShuffle tool: we first benchmark the performance of the uShuffle C library, then compare the performance of the uShuffle Java applet with the shufflet program by Coward [11].

### Performance of uShuffle C Library

We tested the uShuffle C library on a desktop PC^2 ^with test data consisting of both real biological sequences and artificially generated random sequences.

#### Experiment on real biological sequences

The real biological sequences were acquired from two sources: first, 152 protein sequences (with a total of 91262 amino acids) were sampled from the Human Protein Reference Database^3^, one sequence from each of the 152 molecular classes; second, 69 micro RNA precursor sequences (with a total of 4773 nucleotides) of Mus.musculus (house mouse) were extracted from the supplementary data^4 ^of Bonnet et al. [4].

Our experiments on these real biological sequences showed that the uShuffle library is extremely efficient: in just one second, it can generate either (i) 700 doublet-preserving random sequences for each of 152 protein sequences, or (ii) 12000 doublet-preserving random sequences for each of the 69 RNA sequences.

#### Experiments on artificially generated random sequences

In order to analyze the performance of uShuffle with various sets of parameters, we also performed a systematic test of uShuffle on artificially generated random sequences. For simplicity, the sequence lengths were exact powers of two from 2^12 ^to 2^24^, that is, from around 4, 000 to around 16, 000, 000. These numbers are somewhat arbitrary; nothing prevents a user from running uShuffle on very long sequences, even at the genome scale, as long as the computer has enough memory to store the input sequence and has some additional (very minimal, as discussed in the Implementation section) memory required by our implementation.

For each sequence length, 64 uniform random sequences over the English alphabet [a-z] were generated as test sequences; for each test sequence, 64 *k*-let-preserving random sequences were then generated by uShuffle. The total running time for uShuffle to generate the 64 × 64 = 4096 *k*-let-preserving random sequences was recorded for each sequence length. Two getrusage system calls were placed in the test program to sandwich the code region being benchmarked; the differences of the two timestamps were used to calculate the running times.

**Figure 3:**
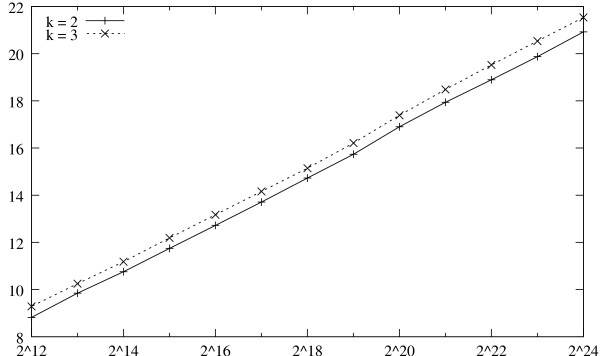
**Running Times of uShuffle for *k *= 2 and *k *= 3**. Running times of uShuffle in milli-seconds at various sequence lengths for *k *= 2 and *k *= 3.

We refer to Figure 3 for a log-log plot of the total running times of the uShuffle program for *k *= 2 and *k *= 3 at various sequence lengths. The plot shows that the running time of the uShuffle program is essentially linear in the length of the sequence to be shuffled.

**Figure 4:**
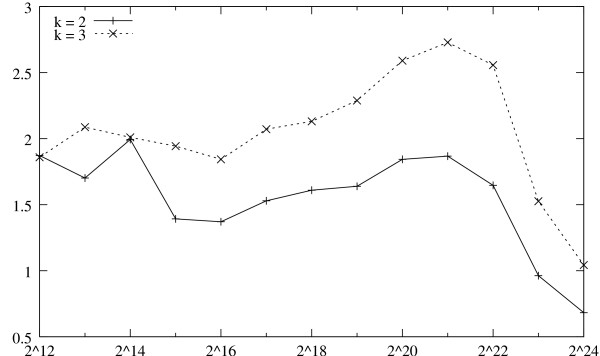
**Ratios of Running Times for *k *= 2 and *k *= 3**. Ratios of running times of uShuffle and simple permutation method at various sequence lengths for *k *= 2 and *k *= 3.

The absolute running times are not very effective in demonstrating the extreme efficiency of the uShuffle program. We refer to Figure 4 for a ratio plot that is more illustrative. For *k *= 2 and *k *= 3, and for each sequence length, the plot shows not the absolute running time of the uShuffle program but the ratio of two running times:

1. the running time for the uShuffle program to generate the *k*-let-preserving sequences, and

2. the running time for the simple permutation method [10] (reviewed in the Background section) to shuffle the same number of random sequences *without *preserving the *k*-let counts.

The ratio plot shows that the running time of the uShuffle program is on average only 1.5 times that of the simple permutation method for *k *= 2, and only 2 times for *k *= 3. The simple permutation method is minimal: for each position of the input sequence it executes only one random function call plus one swap. The uShuffle program, on the other hand, performs a lot more work; although the 64 *k*-let-preserving random sequences of each test sequence are generated by one shuffle1 and 64 shuffle2 function calls to avoid redundant multigraph construction, each shuffle2 function call still includes the generation of an arborescence by loop-erased random walks [19,23] and the generation of an Eulerian walk guided by the individual edge lists shuffled by simple permutations. In light of the contrasting complexities of the uShuffle program and the simple permutation method, the small ratios of their running times are remarkable. A careful reader will notice an interesting fact from Figure 4, when the sequence length increases to 2^24 ^(about 16 millions), the running time of the uShuffle program for *k *= 2 is even *less than *the simple permutation method! The "strange" phenomenon had kept us puzzling for a long time until we eventually convinced ourselves that this is not a bug but a feature. We note that, in each step, the simple permutation method randomly swaps two elements scattered in a large array of 2^24 ^elements. On the other hand, the uShuffle program performs random walks in small multigraphs (at most 26 vertices for *k *= 2 and over the [a-z] alphabet) and permutes the individual edge lists (each with approximately 2^24^/26 elements) separately. The memory references of the uShuffle program are much more *local *than those of the simple permutation method. Computers with modern memory architectures aggressively optimize code with local memory references by sophisticated caching schemes, which promotes the performance of the uShuffle program.

**Figure 5:**
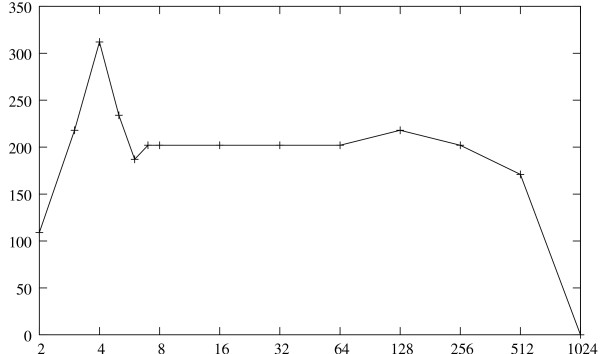
**Running Times of uShuffle for Various Let Size *k***. Running times of uShuffle in milli-seconds at a fixed sequence length for various let size *k*.

We refer to Figure 5 for the running times of the uShuffle program at various values of the parameter *k*, where the test sequence length is fixed at 1024. The running time of the uShuffle program peaks at *k *= 4, which is about three times its running time for *k *= 2, then gradually decreases as *k *increases, and finally drops to zero at *k *= 1024 because, with a sequence length of 1024, the only 1024-let-preserving random sequence is the input sequence itself. This plot shows that the uShuffle program is efficient for all possible values of *k*.

### Comparison of uShuffle Java Applet with shufflet

There exist two other implementations of the Euler algorithm. The dishuffle program by Clote et al. [6] implements the original version of the Euler algorithm by Altschul and Erickson [2]. Hard-coded for shuffling RNA sequences preserving dinucleotide counts, dishuffle is not a general tool for arbitrary alphabet and let sizes. Another program, shufflet by Coward [11], implements the improved version of the Euler algorithm by Kandel et al. [15] for arbitrary let size *k*. As we have explained in the Implementation section, the arborescence generation algorithm by Kandel et al. [15], while superior to the algorithm by Altschul and Erickson [2], is still inferior to Wilson's algorithm [19,23]; besides, its look-up table data structure is inefficient for large alphabet and let sizes.

In terms of functionality, the shufflet implementation [11] is closer to our uShuffle implementation. Shufflet was written in the C programming language, and had been hosted as a web application (but has been taken off-line). We were unable to perform a comprehensive comparison of uShuffle and shufflet. However, Coward [11] mentioned two experiments performed on a Digital DEC/Alpha 2100 web server:

1. 100 shufflings of a DNA sequence of 10000 nucleotides with *k *= 6 take about 2.5 seconds;

2. 100 shufflings of a protein sequence of 1000 amino acids with *k *= 3 take less than 1 second.

We performed similar experiments with the uShuffle Java applet on an Apple iMac computer (2 GHz PowerPC G5 running MacOS 10.4.9, Firefox 2.0.0.3, and Java 1.5.0):

1. 1000 shufflings of a DNA sequence of 10000 nucleotides with *k *= 6 take about 1.5 seconds;

2. 4000 shufflings of a protein sequence of 1000 amino acids with *k *= 3 take less than 1 second.

Assuming comparable performances of the two computers, we estimate that our uShuffle Java applet is about 15–20 times faster than shufflet in the experiment on nucleotides (*k *= 4), and about 40 times faster than shufflet in the experiment on amino acids (*k *= 20).

We certainly understand the difficulty of such a comparison: a web server in 1999 versus a desktop computer in 2005; a C program in a native machine versus a Java applet in a virtual machine. Nevertheless the comparison illustrates the better scalability of our uShuffle Java applet for large let sizes. The difference between the two performance ratios, 15–20 versus 40, suggests that uShuffle remains efficient even for large let size, while shufflet becomes more inefficient as the let size increases, due to (very likely) the use of the inefficient look-up table data structure by Kandel et al. [15].

## Conclusion

The uShuffle tool is based on superior graph algorithms and is carefully engineered to be extremely efficient. It achieves maximum flexibility by allowing arbitrary alphabet size and let size, and is available in many forms for different kinds of users. We believe uShuffle is a useful tool for the bioinformatics community.

## Availability and Requirements

Project name: uShuffle.

Project home page: 

Operating systems: Platform independent.

Programming languages: C, Java, C#, Perl, Python.

Other requirements: None.

Licence: FreeBSD.

Any restrictions to use by non-academics: None.

## Authors' contributions

MJ designed the software and the experiments, implemented the C and Java versions of uShuffle program, and wrote the technical report. JA ported the Java program to C#, investigated software licenses, and designed the uShuffle logo. JG wrote the test program, performed the experiments, and wrote the instructions for Perl integration. MM wrote the Java applet interface and the instructions for Python integration. All authors reviewed the source code and contributed to the home page construction.

## Note

^1^MacOS 10.4.9, Firefox 2.0.0.3, Java 1.5.0.

^2^Dell XPS M1710: 2.1 GHz Intel Dual-Core 2 processor, 2 GB dual in-line RAM; Microsoft Windows Vista Business Edition, Cygwin 1.5.24, gcc 3.4.4 with -O3 option.

^3^.

^4^.
